# Deriving Gene Networks from SNP Associated with Triacylglycerol and Phospholipid Fatty Acid Fractions from Ribeyes of Angus Cattle

**DOI:** 10.3389/fgene.2016.00116

**Published:** 2016-06-20

**Authors:** Justin W. Buchanan, James M. Reecy, Dorian J. Garrick, Qing Duan, Don C. Beitz, James E. Koltes, Mahdi Saatchi, Lars Koesterke, Raluca G. Mateescu

**Affiliations:** ^1^Department of Animal Science, University of California, Davis, DavisCA, USA; ^2^Department of Animal Science, Iowa State University, AmesIA, USA; ^3^Department of Animal Science, University of Arkansas, FayettevilleAR, USA; ^4^Texas Advanced Computing Center, University of Texas at AustinAustin, TX, USA; ^5^Department of Animal Sciences, University of Florida, GainesvilleFL, USA

**Keywords:** beef, SNP, GWAS, fatty acids, gene networks, triacylglycerol, phospholipid

## Abstract

The fatty acid profile of beef is a complex trait that can benefit from gene-interaction network analysis to understand relationships among loci that contribute to phenotypic variation. Phenotypic measures of fatty acid profile from triacylglycerol and phospholipid fractions of longissimus muscle, pedigree information, and Illumina 54 k bovine SNP genotypes were utilized to derive an annotated gene network associated with fatty acid composition in 1,833 Angus beef cattle. The Bayes-B statistical model was utilized to perform a genome wide association study to estimate associations between 54 k SNP genotypes and 39 individual fatty acid phenotypes within each fraction. Posterior means of the effects were estimated for each of the 54 k SNP and for the collective effects of all the SNP in every 1-Mb genomic window in terms of the proportion of genetic variance explained by the window. Windows that explained the largest proportions of genetic variance for individual lipids were found in the triacylglycerol fraction. There was almost no overlap in the genomic regions explaining variance between the triacylglycerol and phospholipid fractions. Partial correlations were used to identify correlated regions of the genome for the set of largest 1 Mb windows that explained up to 35% genetic variation in either fatty acid fraction. SNP were allocated to windows based on the bovine UMD3.1 assembly. Gene network clusters were generated utilizing a partial correlation and information theory algorithm. Results were used in conjunction with network scoring and visualization software to analyze correlated SNP across 39 fatty acid phenotypes to identify SNP of significance. Significant pathways implicated in fatty acid metabolism through GO term enrichment analysis included homeostasis of number of cells, homeostatic process, coenzyme/cofactor activity, and immunoglobulin. These results suggest different metabolic pathways regulate the development of different types of lipids found in bovine muscle tissues. Network analysis using partial correlations and annotation of significant SNPs can yield information about the genetic architecture of complex traits.

## Introduction

Beef is a nutritious source of protein, fat, vitamins, and minerals when appropriately included in the human diet. A large body of research indicates it is critical to maintain a balanced fatty acid intake to support a healthy blood lipid profile (Ooi et al., 2013). The synthesis of adipose tissue in beef cattle is a complex biological process controlled by numerous loci as well as environmental factors. Considerable ongoing effort has been devoted to identification of genomic regions as well as candidate genes for fatty acid profile and adipose synthesis in various breeds of beef cattle, including Angus, Nellore, Brahman, Santa Gertrudis, Hereford, and Shorthorn (Barendse, 2011; Cesar et al., 2014; Kelly et al., 2014). The usefulness of these loci in DNA based beef cattle selection schemes will increase with knowledge of the genomic regions contributing to the development fat deposition (Saatchi et al., 2013).

The fatty acid profile in beef cattle can be characterized by the abundance of 39 individual lipids of varying chain lengths and degrees of saturation (Daley et al., 2010). The total lipids present in animal tissues can be separated into triacylglycerol and phospholipid fractions, which represent the two primary modes of lipid storage in cattle muscular tissue (Yen et al., 2008). The triacylglycerol fraction typically represents 70 to 92% of the total lipid in longissimus muscle depending on age and dietary composition (Warren et al., 2008). The most abundant fatty acids in the triacylglycerol fraction are 18:1, 16:0, and 18:0 (Daley et al., 2010). The fatty acids are primarily derived from the *de novo* synthesis of lipids from the fatty acid synthase (FASN) complex. The most abundant fatty acids in the phospholipid fraction include 18:2, 16:0, and 18:1, with the majority of lipids having at least one unsaturation. Less is known about the origins of these lipid species in phospholipid. Taken together, these data present a large number of phenotypes which are regulated by various networks of genes associated with lipid synthesis and metabolism. Given the increasing availability of SNP data, there has been an interest in developing computational methods that utilize GWAS results from multiple traits along with principles of co-association to identify clusters of SNP that likely regulate the underlying metabolic pathways (Reverter and Chan, 2008; Fortes et al., 2011; Reverter and Fortes, 2013).

Phenotypic correlations among fatty acids within and across lipid fractions have been published (Hoehne et al., 2012), while estimates of phenotypic variance and genetic parameters including correlations have been obtained for the data set used in this study (Buchanan et al., 2015). Following Reverter and Fortes (2013), correlations among multiple phenotypes can be exploited to develop an association weight matrix utilizing high-throughput data such as that obtained from GWAS in order to reveal genomic regions associated with a phenotype or multiple phenotypes of interest. This methodology utilizes a partial correlation and information theory algorithm (PCIT) to analyze an input matrix that contains data from GWAS across multiple phenotypes to generate clusters of loci that are highly associated with the overall trait of interest (Reverter and Chan, 2008). The utility of that analysis has been previously demonstrated through derivation of a regulatory gene network associated with puberty in beef cattle (Fortes et al., 2011). The objective of this study was to utilize the PCIT algorithm to identify clusters of genes affecting variation across 39 different lipid classes from both the triacylglycerol and phospholipid fatty acid fractions in Angus beef cattle.

## Materials and Methods

### Ethics Statement

The Iowa State University and Oklahoma State University Institutional Review Boards approved the experimental protocols used in this study.

### Animals and Sample Collection

A total of 1,833 offspring of 155 Angus bulls represented by bulls (*n* = 450), steers (*n* = 1,022), and heifers (*n* = 361) were used in this study. All cattle were finished on concentrate diets typical for feedlot growth in Iowa (*n* = 908), California (*n* = 344), Colorado (*n* = 291), or Texas (*n* = 290). Animals were harvested at commercial facilities when they reached typical US market endpoints with an average age of 457 ± 46 days. Production characteristics and additional details of sample collection and preparation were reported previously (Garmyn et al., 2011). After external fat and connective tissue were removed, the 1.27-cm steaks from the longissimus muscle were analyzed for fatty acid composition at Iowa State University (Ames, IA, USA). Fatty acids were analyzed using thin layer and gas chromatography to separate the lipid fractions and derivatize to fatty acid methyl esters, respectively. Full details of fatty acid preparation and determination were previously described in detail (Zhang et al., 2008; Buchanan et al., 2015).

### Genome-Wide Association Study of Fatty Acid Fractions

Genomic DNA was extracted from the ground beef sample used for fatty acid composition and was genotyped with the Bovine SNP50 Infinium II BeadChip (Illumina, San Diego, CA, USA). Contemporary groups were defined based on cross-classifications of gender at harvest (bull, steer or heifer), finishing location (California, Colorado, Iowa, Texas), and harvest date, for a total of 33 groups. Contemporary groups were fit as fixed effects in genomic analyses. Effects of SNP on each trait were estimated using the Bayes-B option of GenSel accessed through the BIGSGUI Version 0.9.2 (Kizilkaya et al., 2010). The Markov-chain Monte Carlo approach used to estimate the effect of each SNP involved a 1,000 iteration burn-in period followed by 40,000 iterations used to obtain posterior means of the effect of each SNP. The estimate of the proportion of genetic variation explained by each SNP and each 1 Mb window was obtained for all 39 fatty acid phenotypes for triacylglycerol and phospholipid fractions as 2p(1-p) times the square of the mean effect for that locus^1^. The windowBV yes option was used to form the posterior distribution of genetic variance explained by every 1 Mb window (Garrick and Fernando, 2013).

### Correlations among SNP

Significant pair-wise correlations among SNP effects were calculated across the 39 fatty acid phenotypes using an optimized implementation of the PCIT algorithm. Briefly, this algorithm begins by calculating third-order partial correlation coefficients among all possible trios of SNPs. Within each trio, a direct correlation between any pair of SNPs that does not exhibit a significant partial correlation to the third SNP is considered an independent association, and thus not significant for network co-association. This methodology identifies SNP co-associations that occur more frequently over the range of phenotypes given as input. The full mathematical model for identifying the most co-associated SNPs across multiple phenotypes is explained in detail by Reverter and Chan (2008). The algorithm was optimized to run in parallel at the Texas Advanced Computing Center (Koesterke et al., 2013). For the triacylglycerol fraction an initial set of 454 SNP were selected from the 20 genomic windows of size 1 Mb with the largest posterior probability of association (Garrick and Fernando, 2013) from the 16:0 fatty acid phenotype. The posterior mean SNP effects for these 454 SNP from 16:0 was augmented with the effects from the other 38 fatty acid phenotypes. This 454 × 39 matrix of posterior mean SNP effects was used as the input for the PCIT algorithm to detect co-association of effects for any SNP across multiple fatty acids. All SNP pairs within the matrix were tested for a partial correlation with at least one other SNP in order to create clusters of associated genes. SNP pairs without a significant partial correlation to at least one other SNP were removed from the dataset and not used for subsequent network association analysis since they would appear isolated.

All SNP were selected from 20 genomic windows of size 1 Mb with the largest posterior probability of association from the 16:0 fatty acid phenotype in order to build a matrix of SNP effects for the phospholipid fraction. A vector of posterior mean SNP effects for 418 SNP from 16:0 was augmented with the effects estimated for each of the other 38 fatty acids. PCIT network creation and visualization proceeded identically to the methods described for the triacylglycerol fraction.

Correlations among SNP were used to construct networks of SNP that exhibited common effects across multiple fatty acids. Correlation between SNP pairs with a non-zero partial correlation to another SNP were input into Cytoscape 3.0.2 (Shannon et al., 2003) software to visualize gene network clusters using the MCODE plugin (Bader and Hogue, 2003; Saito et al., 2012). Networks are scored and ranked by the MCODE plugin as network density times the number of nodes. MCODE defines network density as the number of edges in a network divided by the theoretical maximum number of edges. SNP were annotated with the Variant Effect Predictor (VEP) using Bovine UMD 3.1 annotations (McLaren et al., 2010) accessed from the cattle genome analysis data repository (Koltes, 2012).

### Gene Ontology Enrichment Analysis and Visualization

Gene ontology (GO) enrichment was carried out using the DAVID v6.7 Functional Annotation Tool (Huang da et al., 2009a,b) in order to identify biological terms associated with genomic regions and gene networks identified in the GWAS analysis. GO term enrichment and clustering was carried out on all annotated genes within the 20 1 Mb genomic windows associated with the triacylglycerol and phospholipid fractions, respectively. Reported results from the GO term enrichment and clustering include the overall enrichment score, the percentage of genes involved in the given term (%), significance of enrichment or EASE score (*P*-value), fold enrichment (FE), and false discovery rate (FDR). Details of the calculation for each of the parameters associated with the GO term enrichment in DAVID is reported by Huang da et al. (2009b). Ensembl Gene ID’s were extracted from 1 Mb genomic regions from the *Bos taurus* UMD3.1 assembly for use in the GO term enrichment analysis.

## Results and Discussion

### Triacylglycerol Genome-Wide Association Study

The posterior mean estimates for proportion of genetic variance explained by 1 Mb genomic windows as well as the posterior probability of association for selected lipids and lipid classes from the triacylglycerol fraction are shown in **Table 1**. All posterior probabilities of association for the genomic windows displayed in **Table 1** were greater than 97% (*PPA* > 0.97), which indicates a low (<3%) FDR in the model for these windows. This indicates these genomic regions or the regions in neighboring windows likely harbor loci or genomic features that exhibit large effects on the phenotype included in the analysis. Multiple genomic windows were identified which explained between 22.13 and 34.55% of genetic variance for individual lipids found in the triacylglycerol fraction. The genomic window on chromosome 19 at Mb 51 appears to describe a large proportion of the genetic variance across multiple fatty acids in the triacylglycerol fraction, including 14:0, 16:0, 16:1, 18:0, 18:1, saturated fatty acids (SFA), and monounsaturated fatty acids (MUFA). This region harbors the candidate gene FASN, which is known to be associated with primary lipid synthesis in adipose tissue (Zhang et al., 2008; Abe et al., 2009). It follows that this genomic window would explain a large proportion of genetic variance across multiple fatty acids and fatty acid classes due to triacylglycerol functioning as the main storage site for lipids of medium chain length synthesized from FASN (Wood et al., 2008).

**Table 1:** Characterization of top 20 1 Mb genomic windows that account for variation in triacylglyceride lipids.

Trait	Map Pos	RS# Start	RS# End	# of SNP	σ^2^_g_ (%)	PPA
14:0						
	19_51	rs41923412	rs109147235	25	34.55	1.00
	29_18	rs42375315	rs41589183	14	10.65	1.00
	10_19	rs41647457	rs110785951	24	3.21	0.98
16:0						
	17_16	rs109550465	rs110684903	17	22.91	1.00
	7_56	rs41614823	rs42334377	17	16.26	1.00
	19_51	rs41923412	rs109147235	25	16.18	1.00
	1_80	rs43245574	rs110467946	21	10.44	1.00
	16_3	rs41790571	rs41633905	24	9.66	0.99
	29_18	rs42375315	rs41589183	14	5.87	1.00
16:1						
	19_51	rs41923412	rs109147235	25	15.25	1.00
	29_18	rs42375315	rs41589183	14	4.72	1.00
	26_21	rs109309604	rs42086690	20	3.23	1.00
18:0						
	29_18	rs42375315	rs41589183	14	5.95	1.00
18:1						
	19_51	rs41923412	rs109147235	25	22.13	1.00
	8_103	rs109285764	rs41590918	18	6.43	0.97
	16_20	rs110743197	rs42542723	23	5.88	0.99
	29_18	rs42375315	rs41589183	14	4.96	1.00
SFA						
	19_51	rs41923412	rs109147235	25	15.58	1.00
	26_20	rs42981135	rs41623887	21	5.39	0.98
MUFA						
	19_51	rs41923412	rs109147235	25	16.63	1.00


*For each lipid or lipid class the posterior mean of the genetic variance (σ^*2*^_*g*_) explained by the 1 Mb window is given along with window position coordinates (UMD3.1), number of SNP, and posterior probability of association (*PPA*).*

Other genomic regions of significance that appeared in both this data set that separately considers the two fractions and the total fatty acid analysis presented by Saatchi et al. (2013) include windows on chromosome 29 (18 MB) and a region on chromosome 26 (20–21 Mb). The region on chromosome 29 accounts for up to 10.65% of the genetic variance in 14:0, 16:0, 16:1, 18:0, and 18:1. The region on chromosome 26 accounts for up to 5.39% of the genetic variance in 14:0, 16:1, SFA, and MUFA. These regions also harbor candidate genes related to fatty acid synthesis and metabolism including the transcription factor thyroid responsive hormone (THRSP) and the coenzyme stearoyl co-A desaturase (SCD), respectively, as noted by Saatchi et al. (2013). Both of these genes have been previously associated with lipid metabolism in cattle (Graugnard et al., 2010).

Several genomic regions of interest were found to explain relatively large proportions of genetic variance that were not detected in the data presented by Saatchi et al. (2013). A window on chromosome 17 at 16 Mb accounted for 22.91% genetic variance in 16:0. This region harbors the possible candidate gene inositol polyphosphate-4-phosphatase, type II (INPP4B). Cellular localization for INPP4B is in the cytoplasm, and the top Gene Ontology biological process entries for this gene include phospholipid metabolic process, and known associations in bovine indicate a larger role in bone remodeling^2^. No QTL have been reported in the Cattle QTL Database^3^ (Hu et al., 2013) in this region related to fat or fatty acid content. A previously unidentified region on chromosome 8 at 103 Mb accounted for 6.43% genetic variance in 18:1, and a second novel region on chromosome 7 at 56 Mb accounted for 16.26% genetic variance in 16:0. No candidate genes were identified within these regions, nor were they reported in the Cattle QTL Database related to fatness or fatty acid metabolism.

### Phospholipid Genome-Wide Association Study

The posterior mean estimates for genetic variance explained by 1 Mb genomic windows as well as the posterior probabilities of association for selected lipids and lipid classes from the phospholipid fraction are shown in **Table 2**. In contrast to the triacylglycerol fraction, posterior estimates for individual lipids and lipid classes were lower, with only 7 windows displaying a posterior probability of association greater than 90% (*PPA* > 0.90). Windows that explained the majority of genetic variance in lipids from the phospholipid fraction exhibited almost no overlap with those from the triacylglycerol fraction.

**Table 2:** Characterization of top 20 1 Mb genomic windows accounting for variation in phospholipid lipids.

Trait	Map Start	RS# Start	RS# End	# of SNP	σ^2^_g_ (%)	PPA
14:0						
	16_4	rs110257825	rs109105804	26	1.79	0.68
	19_5	rs41633989	rs109106774	17	1.11	0.50
16:0						
	21_36	rs109143576	rs42429437	22	2.54	0.72
	1_52	rs41600017	rs43711327	25	1.10	0.54
16:1						
	4_95	rs43412327	rs42421263	20	1.39	0.68
	X_5	rs109239523	rs29023191	12	0.87	0.55
18:0						
	24_29	rs110012069	rs42837712	24	4.27	0.78
18:2, n-6						
	27_42	rs42135519	rs110741211	25	3.08	0.90
18:3, n-3						
	9_12	rs43731273	rs110226869	21	3.52	0.99
	4_89	rs109964815	rs43586675	24	2.85	0.99
	18_31	rs41574692	rs41581150	11	2.93	0.98
	26_27	rs109158324	rs41636608	18	3.13	0.95
	1_24	rs29011682	rs29017639	27	1.81	0.88
	15_29	rs29010888	rs41582064	25	4.25	0.85
	17_70	rs42794376	rs109349100	21	2.02	0.79
20:0						
	9_54	rs109669651	rs110216218	25	3.18	0.91
	25_6	rs41592046	rs110848072	25	2.04	0.74
	1_73	rs109693922	rs110962722	24	1.82	0.72
20:1						
	9_104	rs41636894	rs41592131	22	3.42	0.93
SFA						
	3_114	rs110764304	rs109271147	30	4.3	0.63


*For each lipid or lipid class an estimate of genetic variance (σ^*2*^_*g*_) explained by the 1 Mb window is given along with window position coordinates, number of SNP, and posterior probability of association (*PPA*).*

Several windows identified harbor candidate genes related to overall lipid and phospholipid metabolism. The genomic window on chromosome 16 at 4 Mb that accounted for 1.79% of the genetic variance in 14:0 harbors the candidate gene fructose-2,6-biphosphatase 2 (PFKFB2). This gene is known to be involved in synthesis and degradation of fructose-2,6-bisphosphate, which is a regulatory molecule involved in glycolysis in eukaryotes (Hue and Rider, 1987). A QTL that spanned this region was identified previously in Angus in relation to 12th rib fat thickness (McClure et al., 2010). Also, the genomic window on chromosome 24 at 29 Mb accounted for 4.27% of genetic variance in 18:0 harbors the candidate gene *N*-cadherin (CDH2). This gene is known to be involved in cell-to-cell adhesion and has been associated with increased adipogenic proliferation in mice (Castro et al., 2004). Other novel genomic windows, including 1 Mb upstream and downstream from the identified regions, did not harbor any candidate genes previously implicated in fatty acid or membrane metabolism.

### Triacylglycerol and Phospholipid Gene Networks

**FIGURE 1:**
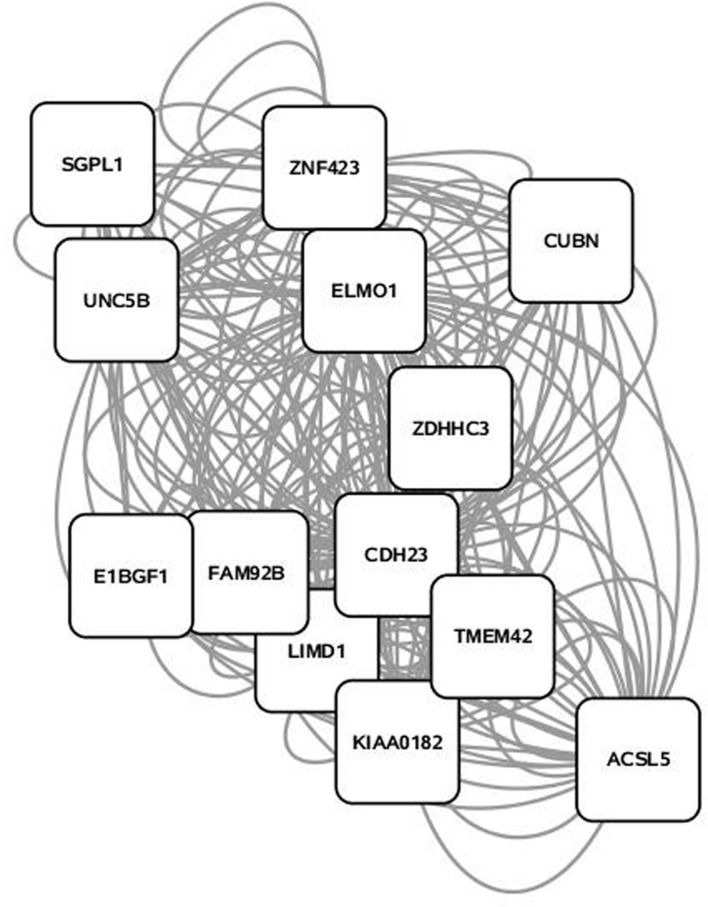
**Highest scoring annotated SNP network from the triacylglycerol fraction derived from PCIT analysis.** Location indicates significance of each node, with distance from the center indicating the number of connections. Each edge represents a correlation identified through PCIT analysis.

**FIGURE 2:**
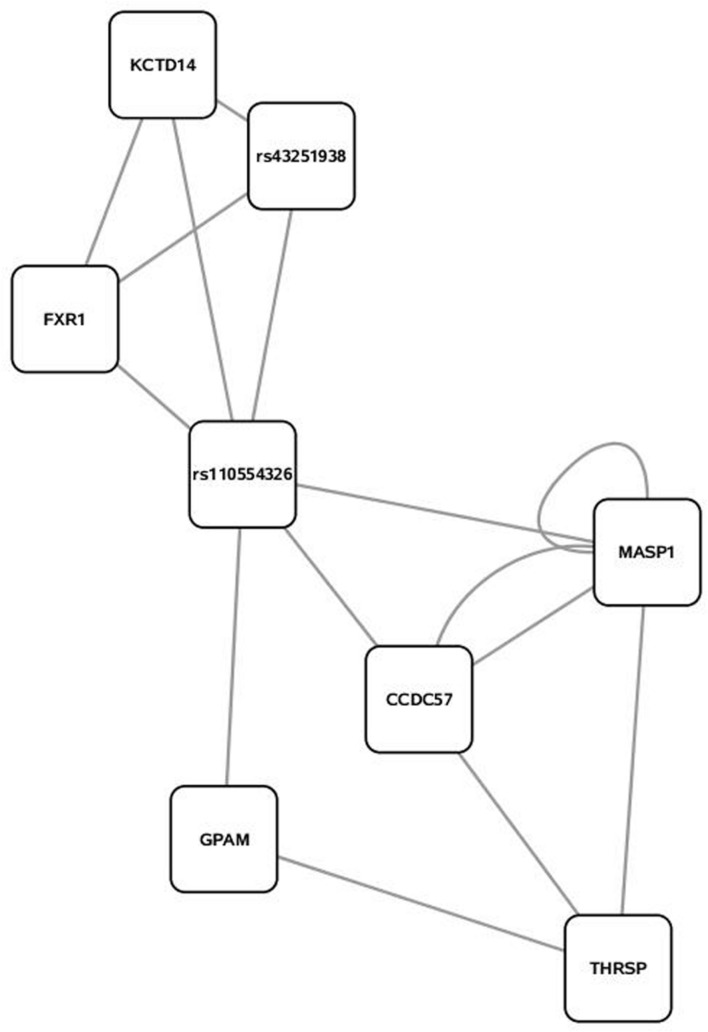
**Second highest scoring annotated SNP network from the triacylglycerol fraction derived from PCIT analysis.** Location indicates significance of each node, with distance from the center indicating the number of connections. Each edge represents a correlation identified through PCIT analysis.

Some 394 of the 454 SNP entered into the PCIT for the triacylglycerol fraction were co-localized into 12 separate networks. Information detailing the top 5 network scoring results from the Cytoscape MCODE plugin for each triacylglycerol-derived network is in **Table 3**. The two highest scoring SNP networks for triacylglycerol fraction obtained from PCIT output and subsequent visualization of nodes with Cytoscape are in **Figures 1** and **2**. Networks are scored and ranked by the MCODE algorithm as network density times the number of nodes. MCODE defines network density as the number of edges in a network divided by the theoretical maximum number of edges. Nodes that were not annotated to a gene or feature were removed from the figures for visual simplicity. Location within the network indicates significance of each node, with greater distance from the center indicating a lower number of overall connections and importance to the phenotype. Each edge represents a connection, or direct correlation, identified through PCIT analysis. **Figure 1** displays the highest scoring triacylglycerol annotated sub-network (un-annotated markers removed) from scoring with the Cytoscape MCODE plugin. The highest scoring network contained 56 nodes and 1487 edges, or connections. **Figure 2** displays the second highest scoring annotated triacylglycerol network obtained with MCODE, which contained 92 nodes and 1699 edges. The clusters of genes represent scored networks derived using PCIT that function as molecular complexes related to the input phenotype. The full output including marker lists for each network from the MCODE analysis for both the triacylglycerol and phospholipid fractions is included in Supplementary Data S1.

**Table 3:** MCODE results derived from network clustering with PCIT.

Fraction	Network	Score	Nodes	Edges
Triacylglycerol				
	1	53.26	56	1487
	2	37.24	92	1699
	3	23.56	58	559
	4	19.26	78	662
	5	18.18	38	278
Phospholipid				
	1	33.91	52	987
	2	29.40	65	1034
	3	26.10	82	1064
	4	10.00	66	468
	5	7.40	51	365


*Top 5 network scores from MCODE plugin for triacylglycerol and phospholipid fractions. Network score represents density of nodes and edges in each network.*

Candidate genes involved in fatty acid metabolism found within these networks include THRSP, Acyl-CoA synthetase-5 (ACSL5), glycerol-3-phosphate acyltransferase muscle-type (GPAM), and coiled coil domain-containing 57 (CCD57). The candidate genes THRSP and GPAM have been previously identified as playing a role in lipid metabolism in beef and dairy adipose via the PPAR pathway (Graugnard et al., 2010; Ji et al., 2014; Moisa et al., 2014). The enzyme ACSL5 is found primarily in cells with a high triacylglycerol synthesis activity, which indicates a likely role in development of adipose (Bu and Mashek, 2010). CCD57 is known to be involved in DNA binding and regulation of gene expression. This gene is located next to FASN on chromosome 19, and has been previously associated with 14:0 content and transcripts have been detected in excess of FASN transcripts in second-lactation dairy cattle (Bouwman et al., 2014; Canovas et al., 2014). Overall, this methodology presents strong evidence that biologically relevant genes can be co-localized with a close relationship to triacylglycerol variation and assembly.

**FIGURE 3:**
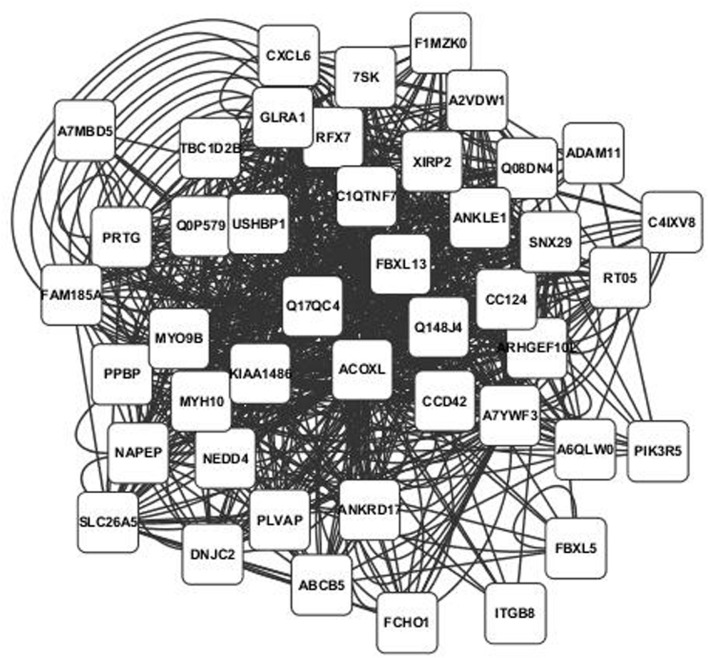
**Highest scoring annotated SNP network from the phospholipid fraction derived from PCIT analysis.** Location indicates significance of each node, with distance from the center indicating the number of connections. Each edge represents a correlation identified through PCIT analysis.

**FIGURE 4:**
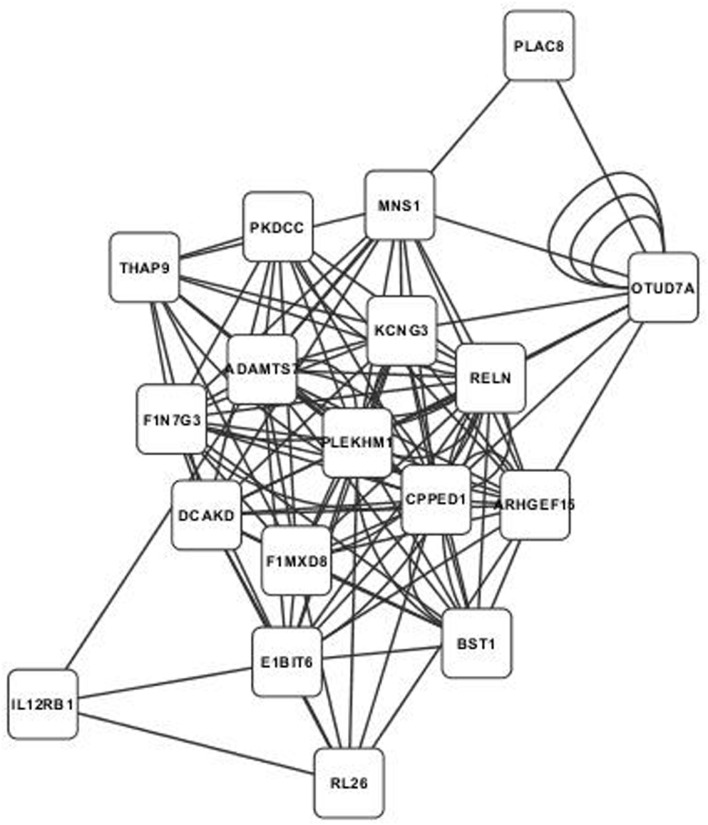
**Second highest scoring annotated SNP network from the phospholipid fraction derived from PCIT analysis.** Location indicates significance of each node, with distance from the center indicating the number of connections. Each edge represents a correlation identified through PCIT analysis.

Annotated visualizations of the two highest scoring phospholipid networks with Cytoscape are in **Figures 3** and **4**. The network in **Figure 3** is the highest scoring phospholipid network containing 52 nodes and 987 edges. **Figure 4** displays the second highest scoring phospholipid network which contained 65 nodes and 1,034 edges. Multiple genes annotated within these networks are known to be involved in cellular trafficking and cell integrity functions associated with the phospholipid membrane. Multiple genes that are known to be involved in membrane binding and cellular trafficking were identified including myosin-IXB (MYO9B), FCH domain only protein 1 (FCHO1), and ADAM metallopeptidase domain (ADAM11). Overall, there were relatively fewer genes identified in the phospholipid fraction analysis that were annotated to genes involved in membrane and lipid metabolism. This difficulty in identifying associated genes could be partly due to the low variance observed in those phenotypes.

### Gene Ontology Term Enrichment Analysis

Gene ontology term enrichment analysis was carried out for all genes located in the top 1 Mb regions shown in **Tables 1** and **2** using DAVID Functional Classification Clustering tools. Genes were obtained by extracting ENSEMBL Gene ID features from the regions of interest. Significant results for the DAVID Functional Annotation Clustering and Functional Annotation Chart results for the top GWAS regions for both fractions are in **Table 4** through **Table 7**. The top GO term clusters for the top 1 Mb windows associated with the triacylglycerol fraction are in **Table 4**. DAVID Functional Annotation Clusters are considered significant above an enrichment score of 1.3. In the functional annotation chart GO terms are considered significantly enriched at a *P*-value of 0.05 or less (Huang da et al., 2009b). The FDR is provided in the Functional Annotation Chart to determine emphasis to be placed on terms considered significant through the *P*-value statistic.

**Table 4:** DAVID Functional Annotation Clustering for significant 1 Mb windows identified through genome-wide association studies of the Triacylglycerol fraction.

Annotation Cluster 1	Enrichment Score: 1.71				
Category	Term	Count	%	*P*-value	FE
GOTERM_BP_FAT	GO:0048872∼homeostasis of number of cells	6	4.32	<0.01	11.52
GOTERM_BP_FAT	GO:0042592∼homeostatic process	8	6.12	<0.01	2.96
GOTERM_BP_FAT	GO:0030099∼myeloid cell differentiation	3	2.54	0.04	8.49
GOTERM_BP_FAT	GO:0048534∼hemopoietic or lymph organ dev.	6	2.65	0.05	6.02
GOTERM_BP_FAT	GO:0002520∼immune system development	4	2.84	0.06	4.56
GOTERM_BP_FAT	GO:0030097∼hemopoiesis	4	3.65	0.18	3.25

**Annotation Cluster 2**	**Enrichment Score: 1.59**				
**Category**	**Term**	**Count**	**%**	***P*-value**	**FE**

SP_PIR_KEYWORDS	nadp	5	2.32	<0.01	9.25
SP_PIR_KEYWORDS	nad	6	4.17	<0.01	6.54
INTERPRO	IPR016040:NAD(P)-binding domain	6	3.21	0.02	5.66
UP_SEQ_FEATURE	active site: Proton acceptor	6	5.84	0.02	4.57
SP_PIR_KEYWORDS	oxidoreductase	8	5.65	0.02	1.99
GOTERM_BP_FAT	GO:0055114∼oxidation reduction	3	6.98	0.03	2.26


*Statistics associated with GO terms include percentage of genes involved in the given term (%), significance of enrichment or EASE score (*P*-value), and fold enrichment (FE).*

The full list of GO terms is displayed in the Functional Annotation Chart results in **Table 5**. Two clusters were produced for the triacylglycerol fraction with an enrichment score above 1.3. Significant GO terms featured in the Functional Clusters and Functional Annotation Chart included homeostatic process (GO:0042592), homeostasis of number of cells (GO:0048872), dendrite development (GO:0016358), and activation of MAP kinase activity (GO:0000187). These terms appear to be associated with features relating to cellularity and energy homeostasis pathways, which have relevance to fatty acid deposition and adipogenesis. The link between adipose tissue cellularity and fatty acid profile has been previously established (Costa et al., 2012). These results highlight the genetic involvement of cellular homeostasis in triacylglycerol metabolism.

**Table 5:** DAVID Functional Annotation Chart results for significant 1 Mb regions identified through genome-wide association studies of the Triacylglycerol fraction.

Category	Term	Count	%	*P*-value	FE	FDR
GOTERM_BP_FAT	GO:0048872∼homeostasis of number of cells	6	3.18	<0.01	11.12	1.08
SP_PIR_KEYWORDS	nadp	5	3.32	<0.01	9.35	3.06
SP_PIR_KEYWORDS	nad	6	4.56	<0.01	6.44	3.50
GOTERM_BP_FAT	GO:0042592∼homeostatic process	9	6.48	<0.01	2.66	4.70
GOTERM_BP_FAT	GO:0016358∼dendrite development	4	2.20	<0.01	25.19	5.35
GOTERM_BP_FAT	GO:0000187∼activation of MAPK activity	3	2.40	0.01	17.56	15.71
INTERPRO	IPR016040:NAD(P)-binding domain	5	3.51	0.02	5.03	12.45
GOTERM_CC_FAT	GO:0016607∼nuclear speck	3	2.95	0.02	12.48	18.97
UP_SEQ_FEATURE	active site:Proton acceptor	5	4.65	0.02	4.15	20.14
GOTERM_BP_FAT	GO:0043406∼positive regulation of MAP kinase activity	3	2.32	0.02	12.53	36.20
KEGG_PATHWAY	bta04130:SNARE interactions in vesicular transport	5	2.65	0.03	9.26	45.25
GOTERM_BP_FAT	GO:0043113∼receptor clustering	3	1.43	0.03	45.62	32.65
GOTERM_CC_FAT	GO:0043233∼organelle lumen	6	5.85	0.03	2.35	32.54
GOTERM_BP_FAT	GO:0030099∼myeloid cell differentiation	3	2.45	0.03	6.95	39.56
GOTERM_CC_FAT	GO:0005739∼mitochondrion	3	6.84	0.03	3.62	38.32
GOTERM_BP_FAT	GO:0007172∼signal complex assembly	2	2.10	0.03	59.63	47.65
GOTERM_CC_FAT	GO:0031974∼membrane-enclosed lumen	8	6.56	0.03	3.26	39.25
SP_PIR_KEYWORDS	Chaperone	4	2.78	0.04	5.64	42.33
GOTERM_BP_FAT	GO:0043405∼regulation of MAP kinase activity	3	2.18	0.05	7.75	49.62
GOTERM_CC_FAT	GO:0016604∼nuclear body	3	2.86	0.05	8.56	45.25
SMART	SM00241:ZP	2	1.62	0.05	26.69	36.21
UP_SEQ_FEATURE	nucleotide phosphate-binding region:NAD	3	2.32	0.05	7.56	35.26


*Statistics associated with GO terms include percentage of genes involved in the given term (%), significance of enrichment or EASE score (P-value), fold enrichment (FE), and false discovery rate (FDR).*

Functional Annotation Clustering analysis for the top GWAS regions associated with phospholipid is in **Table 6** and the full list of significant GO terms from the Functional Annotation Chart is in **Table 7**. Only one significant GO term cluster was identified for phospholipid regions with an annotation cluster score above 1.3. Top significant enriched GO terms included protein serine/threonine kinase activity (GO:0004674), immunoglobulin mediated immune response (GO:0002455), and plasma protein inflammatory response (GO:0002541). There is evidence for a link between prolonged protein kinase activation and a cellular signaling cascade that may result in the degradation of lipid membrane constituents (Nishizuka, 1995). However, without further evidence it is not immediately apparent how this term enrichment relates to lipid membrane metabolism. There also appear to be a large number of enriched terms associated with immune response pathways, suggesting a possible role in unsaturated lipid signaling in these processes.

**Table 6:** DAVID Functional Annotation Clustering for significant 1 Mb regions identified through GWAS for the Phospholipid fraction.

Annotation Cluster 1	Enrichment Score: 1.38				
Category	Term	Count	%	*P*-value	FE
INTERPRO	IPR013783:Immunoglobulin-like fold	6	7.82	<0.01	6.32
INTERPRO	IPR007110:Immunoglobulin-like	8	6.94	<0.01	4.98
INTERPRO	IPR013106:Immunoglobulin V-set	3	5.02	0.01	7.84
SP_PIR_KEYWORDS	Immunoglobulin domain	5	5.19	0.01	7.89
INTERPRO	IPR003599:Immunoglobulin subtype	4	3.56	0.13	5.62
SMART	SM00409:IG	5	4.45	0.18	3.21
UP_SEQ_FEATURE	glycosylationsite: N-linked (GlcNAc…)	6	8.95	0.20	2.12
SP_PIR_KEYWORDS	glycoprotein	9	7.56	0.58	2.31


*Statistics associated with GO terms include percentage of genes involved in the given term (%), significance of enrichment or EASE score (P-value), and fold enrichment (FE).*

**Table 7:** DAVID Functional Annotation Chart results for significant 1 Mb regions identified through GWAS for the Phospholipid fraction.

Category	Term	Count	%	*P*-value	FE	FDR
INTERPRO	IPR013783:Immunoglobulin-like fold	9	9.21	<0.01	6.32	1.22
INTERPRO	IPR007110:Immunoglobulin-like	5	8.45	<0.01	6.56	4.56
INTERPRO	IPR013106:Immunoglobulin V-set	6	6.54	0.01	9.01	10.10
SP_PIR_KEYWORDS	Immunoglobulin domain	4	3.98	0.02	9.65	11.89
GOTERM_CC_FAT	GO:0048475∼coated membrane	4	3.68	0.02	11.25	19.56
GOTERM_CC_FAT	GO:0030117∼membrane coat	4	2.99	0.03	12.45	21.54
INTERPRO	IPR000920:MyelinP0 protein	3	3.21	0.03	78.65	24.82
GOTERM_BP_FAT	GO:0046907∼intracellular transport	6	6.32	0.03	5.05	35.18
GOTERM_MF_FAT	GO:0000166∼nucleotide binding	11	11.25	0.03	2.03	36.32
GOTERM_CC_FAT	GO:0030663∼COPI coated vesicle membrane	3	3.21	0.04	44.52	35.02
GOTERM_CC_FAT	GO:0030126∼COPI vesiclecoat	2	2.65	0.04	42.17	35.26
GOTERM_CC_FAT	GO:0030137∼COPI-coated vesicle	2	2.32	0.05	43.65	39.15
GOTERM_BP_FAT	GO:0006886∼intracellular protein transport	4	4.84	0.05	5.89	54.55


*Statistics associated with GO terms include percentage of genes involved in the given term (%), significance of enrichment or EASE score (*P*-value), fold enrichment (FE), and false discovery rate (FDR).*

## Conclusion

Analysis of GWAS results for triacylglycerol and phospholipid fractions supports the conclusion that the triacylglycerol fraction is closely representative of the total fatty acid fraction. Significant genomic windows identified for triacylglycerol fraction overlapped with the results previously presented by (Saatchi et al., 2013) based on total fatty acids in the same samples. This is expected since the phenotypic measurements of the predominant triacylglycerol fraction are similar to those for the total fatty acids. It follows that the genomic regions that likely harbor genes and features related to the total fatty acids would also be identified in the triacylglycerol. The triacylglycerol fraction also exhibits a much larger genetic variance when compared to the phospholipid fraction. This methodology can yield candidate markers associated with intramuscular adipose accumulation as well as an enriched set of biological functions representative of fatty acid deposition in beef cattle.

An analysis of the genomic regions that affect the phospholipid fraction yielded few genes with a known biological association to lipid metabolism. Significant genomic regions identified explained lower percentages of genetic variance in comparison to the triacylglycerol. The low variation observed in the phospholipid fraction is likely due to the importance of the phospholipid membrane to biological function of the cell. Pathways prevalent in the phospholipid analysis appeared to be highly related to cell-to-cell adhesion, cellular trafficking, and coenzyme/cofactor activity. A larger dataset could possibly improve results when dealing with traits that exhibit low phenotypic variance. In conclusion, the combination of GWAS results with PCIT and network visualization represents a robust methodology for identifying candidate genes of interest for traits with multiple phenotypes and adequate phenotypic variance.

## Author Contributions

JB conducted the analysis and drafted the manuscript. JR conceived the analysis and assisted with the manuscript. DG assisted with the analysis and manuscript. QD assisted with data collection and analysis. DB assisted with he analysis and the manuscript. JK conceived the analysis and assisted with the manuscript. MS conceived the analysis and assisted with the manuscript. LK assisted with the methodology and and analysis. RM conceived the analysis and assisted with the manuscript.

## Conflict of Interest Statement

The authors declare that the research was conducted in the absence of any commercial or financial relationships that could be construed as a potential conflict of interest.

## Footnotes

1 http://www.biomedcentral.com/content/supplementary/1471-2105-12-186-s1.pdf

2 http://amigo.geneontology.org/amigo

3 http://www.animalgenome.org/cgi-bin/QTLdb/BT/index
